# Occupational Therapists' Career Planning, Development, and Progress: An Australian Mental Health Perspective

**DOI:** 10.1155/oti/3901634

**Published:** 2025-02-26

**Authors:** Gemma Keep, Rosalind Bye, Clyde Eriksson, David Lim

**Affiliations:** ^1^School of Health Sciences, Western Sydney University, Campbelltown, New South Wales, Australia; ^2^Older Persons Community Mental Health Team, Nepean Blue Mountain Local Health District, Penrith, New South Wales, Australia; ^3^Faculty of Health, University of Technology Sydney, Broadway, New South Wales, Australia

**Keywords:** career choice, career mobility, health workforce, mental health, occupational therapists

## Abstract

**Introduction:** Mental health occupational therapy is an expanding workforce due to the increasing demand for services in many countries, including Australia. Due to the nature of the role, therapists can experience unique challenges that impact retention and wellbeing, consequently affecting career progression. It is, therefore, important to understand career planning and development for mental health occupational therapists to ensure their professional needs are addressed, which would also benefit service sustainability. This study is aimed at understanding the perspectives of mental health occupational therapists within the Nepean Blue Mountains Local Health District on career planning, development, and progression. This health district spans urban and regional areas and has recently introduced a new career framework underpinned by Benner's career planning model. The practical implications of this study are significant, as the insights gained will inform the development of strategies and policies that support the career progression and wellbeing of mental health occupational therapists, ultimately enhancing the quality and sustainability of service provision in this field.

**Method:** In this qualitative descriptive study, seven occupational therapists shared their perspectives through semistructured interviews. The data were transcribed verbatim, and an inductive qualitative content analysis was employed.

**Results:** Three categories and seven subcategories were identified. The health district career framework was found to be beneficial to support career planning and development, with a few adjustments suggested by participants.

**Conclusion:** Having a career framework offers a structured approach to support career planning, development, and progression for mental health occupational therapists.

## 1. Introduction

Mental health service provision is a core role for occupational therapists (OTs) [1]. OTs working in mental health (hereby respectfully referred to as mental health OTs, whilst acknowledging that not all countries have defined specialist scope or protected terms for mental health OTs) meet diverse consumer needs and work in various treatment settings and service types, meaning a large workforce is required to meet current and future consumer demands. According to the Australian Institute of Health and Welfare [2], there were 2605 mental health OTs working in Australia in 2021. This encompassed nearly 12% of the occupational therapy workforce [3]. With over 1 in 5 Australians experiencing mental health concerns [4], there is a need to ensure the mental health occupational therapy workforce is supported in a sustainable way to assist this important area of practice.

Several studies have investigated the challenges mental health OTs experience as part of their role. A study examining survey responses from 63 OTs working in youth mental health in Australia found they had larger caseloads and limited support from management, which impacted on continuing professional development and evidence-based practice [5]. Another Australian study of mental health OTs indicated that they were at greater risk of experiencing burnout, low work engagement, and poor job satisfaction [6]. These factors were linked with poor work-related wellbeing in another study by Scanlan, Meredith, and Poulsen [7], thus increasing the likelihood of therapists wanting to leave their role. High staff turnover was linked with a loss of organisational knowledge, compromised continuity of care, and interruptions in evidence-based practice [7]. Additionally, within a multidisciplinary mental health team, it is common for the role of OTs to be poorly understood [8]. In Wan Yunus et al.'s study (2022), OTs in Malaysia needed to continually promote the profession's role to maximise client outcomes. The extra workload, poor staff retention, and lack of understanding and appreciation of the occupational therapy role can consequently impact mental health OTs' careers.

To date, less attention has been paid to occupational therapy career planning, development, and progression in general. What has been noted in an Australian study by Nelson et al. [9], who conducted semistructured interviews with 11 purposefully selected OTs, is that career planning is not a linear or sequential process. Instead, OTs' readiness to progress was influenced by supervision, informal networking, guidelines, and expectations for their new position [9]. Additionally, an editorial by Craik [10] addressing the career progression of OTs in the United Kingdom noted that secondments, widely used in health contexts, were beneficial to facilitate skill development to progress to the next career stage. Craik [10] recommended that all OTs actively plan and develop their careers to ensure adequate progression to meet aspirations.

Research on occupational therapy career planning, development, and progression is limited in the mental health sector. Some time ago, Scanlan et al. [11] identified the need for future research to better understand the mental health occupational therapy workforce and develop approaches to support careers. A more recent study by Foster, Palexas, and Hitch [12] reviewed current practices for mental health occupational therapy early career programs in Victoria, Australia, and found that debriefing, self-care skills, reflective practice, and formalised performance and development plans were key factors influencing career progression. Considering the need to retain clinicians to meet growing demands from consumers and services, career planning, development, and progression-related research is a priority for this area.

The Nepean Blue Mountains Local Health District (NBMLHD) is located approximately 50 km west of the Sydney central business district, servicing a large, culturally diverse community of some 384,742 residents across both urban and regional areas comprising some 9179 km^2^. In the early 2020s, a career pathway framework known as the “Allied Health Career Pathways Guide” (available from authors on reasonable request) was developed to attract, recruit, and retain high-quality allied health professionals, including OTs, and support existing staff in their professional development journey [13]. The career pathway guide was developed using Benner's novice-to-expert model [14] and outlines five levels of career progression: novice, beginner, adept, advanced, and senior specialisation [13]. Benner's (1982) model has predominantly been utilised to address career progression for nursing but was recently adopted by the Welsh Government to map out its allied health career framework [15]. To date, there is limited research regarding the use of Benner's model in occupational therapy career planning.

The NBMLHD framework is aimed at assisting allied health staff in identifying essential knowledge and skills for each level, strengthening and supporting workplace practices, and increasing job satisfaction [13]. Collectively, these processes are aimed at contributing to a competent and engaged allied health workforce within the NBMLHD. The NBMLHD mental health occupational therapy team proposed and coproduced this study to explore how the new framework aligned with the perceptions of mental health OTs regarding their career planning, development, and progression. The research questions guiding this study were as follows: (1) what are mental health OTs' perspectives regarding their career planning, development, and progression and (2) how might the new NBMLHD framework influence this process?

## 2. Methods

Under a social constructivist paradigm [16], this study utilised a qualitative descriptive approach [17] to explore mental health OTs' perspectives. This approach is aimed at describing the perspectives under investigation and providing a comprehensive summary of individuals' unique contexts [18]. Additionally, this study was conceptually informed by the NBMLHD career framework and Benner's model [13, 14]. These conceptual models shaped the study by enhancing the researchers' understanding of career planning, development and progression, and informing the interview questions.

Ethical approval was obtained by both Western Sydney University and NBMLHD prior to commencing this study (H15422 and ETH00517).

All NBMLHD mental health OTs (*N* = 18) were invited to participate in this study. Mixed purposive sampling [19, 20] was utilised to recruit participants. Recruitment invitations were emailed from the university researchers to avoid any coercion from the NBMLHD research team member. Participation information sheets were provided and informed written consent was obtained prior to engaging in the study. Participation was voluntary, and participants were informed they could withdraw from the study before the member-checking process was finalised and data was deidentified.

Seven therapists agreed to participate. All seven were Level 3 or higher under the New South Wales public sector allied health employment award [21], meaning they were experienced clinicians with extensive knowledge within their discipline (please refer to the Supporting Information section (available here) for classification of allied health levels). Participants had a mean of 10.7 years of experience working as OTs. Further demographic data was deemed potentially identifiable given that the participants were drawn from a known distinct pool, and therefore, this information is not included in the reporting.

### 2.1. Data Collection

Data collection involved a single semistructured interview conducted by G.K. and D.L. The interview guide, which included information about how their careers progressed and what influenced this process, was utilised. It was informed by the systematic review currently under review and codesigned with the NBMLHD research team.

Participants chose the format, with five electing face-to-face interviews and two choosing an online Microsoft Teams interview. Interviews ranged in length from 30 to 50 min and were audio recorded. The research student, G.K., completed reflection journal entries after each interview to summarise the main themes. Interviews were transcribed verbatim, and participants were given the option to undertake member checking [22, 23]. Participants were emailed their transcripts within 7 days to review and return to the researcher. Transcripts were deidentified before analysis. Preliminary analyses were shared with participants for validation and offered further opportunities to provide additional data. No additional data were forthcoming at the validation stage.

### 2.2. Data Analysis

Inductive qualitative content analysis was adopted for this study to enable a description of data close to participants' own words [24]. Inductive qualitative content analysis is a process by which categories are derived from the data and has three distinct phases [25]. The preparation phase involved the student researcher immersing in the data through repeated readings of transcripts and reflection entries. After making sense of the data, the organisational phase began. This phase involved breaking down the transcripts into meaning units, which were a few sentences in length, and labelling them with a code to add to a coding list. As the coding list was generated inductively, it was altered as more data became available. The coded data was then condensed into subcategories to describe the data. Subcategories were grouped into categories, and categories were grouped into an overarching category. This abstraction of categories was completed several times until a reasonable descriptive explanation was achieved to answer the research questions [25]. Finally, the reporting phase provided a detailed description of the categories identified in the organisational phase.

### 2.3. Rigour

This study's rigour was maintained using four criteria for the trustworthiness of qualitative research outlined by Lincoln and Guba [26]: credibility, dependability, confirmability, and transferability. Member checking and peer review were utilised to ensure analytical agreement between researchers and participants. Interview transcripts were examined by a third researcher, R.B., who was not involved in the interview and transcription process to maintain dependability. Information on the setting, analysis process, and verbatim quotes were provided in the reporting of results to ensure the credibility and transferability of the study.

## 3. Results

Data analysis revealed one overarching category: mental health occupational therapy career progression is multifaceted. The findings were organised into categories and subcategories underneath this overarching category, as outlined in Table 1. These categories, illustrated by quotes, reveal how OTs working in the NBMLHD mental health service perceived career planning and development and their newly proposed career framework.

### 3.1. Mental Health Occupational Therapy Career Progression Is Multifaceted

The overarching category that emerged from the data was that career progression for mental health OTs within the study context was multifaceted. As Participant 3 explained, “You can progress as a clinical specialist, you can progress into a management pathway, and you can also do research and education.”

In addition to different pathways, there were also increased role opportunities in mental health through the ability to work in occupational therapy-specific roles or roles that were nondiscipline-specific. Participant 4 noted, “there's a lot more opportunity for occupational therapists to grow and work their way up in mental health because there are more management roles, not necessarily occupational therapy specific roles”. However, starting an occupational therapy career in a nonoccupational therapy-specific role was perceived as potentially reducing a novice therapist's occupational therapy skill set and impacted professional identity. Participant 6 stated, “I felt that my skills got diluted quite quickly because I didn't really have an occupational therapist to support me early on in developing my … skills”.

External influences, such as the variety of available mental health roles, added to the range of opportunities for progression. Participant 1 discussed how mental health “deals with the entire lifespan,” with the option to work with children and adolescents, adults, and older people, with multiple roles available for each age group of clients.

With the different pathways available to develop a career in mental health, either in an occupational therapy role or a more generic position, participants reported less structure in progressing through the NBMLHD's allied health award levels compared to other practice areas. This could be an advantage as junior therapists can move ahead quickly in their careers, especially between allied health award Levels 1 and 2. Participant 2 discussed this opportunity:

There is lots of opportunity to move ahead quickly. It's a little less structured in that sense. It's more about your competence and your confidence, whereas some of the other clinical areas they care about how many years you have been working.

Whilst the “many right doors” situation may be beneficial, participants also highlighted the challenging nature of mental health roles and how that impacted navigating allied health career levels in the relevant award structure. For therapists wanting to remain clinically focused, this could be more challenging. Participants discussed how remaining clinically focused limited career progression as fewer higher-level clinical roles were available. The award structure in place in the NBMLHD meant that becoming more senior also involved the requirement to undertake leadership roles, either as a professional lead with recognised clinical expertise or by managing a team.

I know some occupational therapists that have been on level two/four for 15-20 years … I find that's what you need to do if you just want to focus on clinical work. As soon as you go into that level three there's this expectation around leadership skills around providing back to the broader profession across the service. (Participant 1)

Another factor impacting career progression on a wider level is the “competitive recruitment process” (Participant 6) of the local health district. Participants discussed a shift in job vacancies since coming to the local health district, as fewer senior roles were available. Participant 5 said, “I've observed within the district … there'll be quite a few occupational therapists that want to progress … so it can be a bit competitive at times to get those senior roles”. This can impact career progression and retention of staff.

As such, participants described a multifaceted context shaping their career planning, development, and progression. They specifically noted aspects that influenced their career journey. These are presented below in the three categories: influences on the choice of a mental health career, influences on career progression, and perception of the new career framework introduced by the local health district.

#### 3.1.1. Influences on the Choice of a Mental Health Career

Within this broader category, participants discussed two main factors influencing their choice of a mental health career: (a) passion for working in mental health and (b) a positive placement experience.

##### 3.1.1.1. Passion for Working in Mental Health

Many OTs expressed a deep passion for working in mental health. They found this area rewarding, with the ability to holistically support consumers across the lifespan. “There's such a broader range of interventions you can do with someone in all areas of their life … I just love that and love being able to look at all aspects of a person” (Participant 5).

The passion for mental health started before commencing their occupational therapy education for some participants. Participant 3 noted, “I was fascinated as early as a 15–16-year-old” in the mental health area. Others found their passion during university education, with Participant 6 commenting, “we had two mental health units and I really came away from them feeling really engaged in the content. So that was probably the main reason that I pursued it”. A passion for this area of practice influenced participants' choices and led them to choose a career in mental health.

A pattern also emerged in participants' perspectives where they noted once they entered the mental health area of practice, their love and passion for the work solidified, with one noting, “I think once you're in mental health, if you love it, you love it” (Participant 4). As such, participants' interest and engagement in a mental health career was reinforced through a love of the work and was a driving factor in choosing and remaining in the mental health area of practice.

##### 3.1.1.2. A Positive Placement Experience

Most participants discussed that a positive undergraduate practice placement in mental health was transformative in their eventual choice of practice area. Many stated prior to their mental health university placement, they did not want to enter the mental health field, but their views changed after having a positive placement experience. “I foresaw mental health as being something I wanted to do from my first placement … I knew from that placement, I definitely wanted to do mental health” (Participant 4).

A positive placement experience exposed participants to opportunities as an OT in this area of practice and was pivotal in influencing their career choices. If the experience was positive, it increased their understanding of how OTs work in this area and encouraged them to seek a career in mental health in the future.

#### 3.1.2. Influences on Career Progression

Within this broader category, therapists discussed three main subcategories that influenced career progression: (a) secondments, (b) supervision and management, and (c) individual traits.

##### 3.1.2.1. Secondments

Secondment opportunities allowed participants to act in a position outside their usual role for a short period of time before returning to their previous role. In most instances, secondments were placed in higher-level positions in the health district. Some participants found the experience beneficial as it allowed them to progress more quickly and develop new skills to secure higher, more competitive roles after the secondment, as noted by Participant 5: “The opportunity to step into roles temporarily was a huge part of my development.”

However, other participants found that if these short-term higher level secondments meant they also had to juggle their current role simultaneously, a challenging experience could result.

I've acted in positions but still held responsibilities from other positions at the same time … it does place challenges and increasing demands on the people who are trying to have a different opportunity and fulfil a different role, but whilst also trying to keep another role afloat at the same time. (Participant 3)

Additionally, participants could be discouraged when secondments to a higher level did not have the guarantee of a full-time position after completing the short-term contract. Some participants could not pursue secondments, despite their interest, as their current role did not have the flexibility to release them to engage in the opportunity. This could be discouraging for those keen to progress within the local health district. Participant 7 explained the nature of these opportunities by noting that

A big barrier [is] when you are short staffed, you cannot release staff to go and act in positions. So you would have to resign to then move into something else … that's a pretty daunting thing and would probably stop a lot of people trying new roles.

Therefore, secondment opportunities may be both positive and negative, depending on the wider team, staffing levels, and timing.

##### 3.1.2.2. Supervision and Management

Supervision provided by a more experienced clinician was key to facilitating career progression. It provided the supervisee with guidance and resources around skill development, knowledge consolidation, and securing different opportunities, as explained by Participant 1 who noted, “supervision was a really helpful resource to be able to gain skills, pathways, and knowledge to be able to then progress to a senior position.”

Some participants reported having a manager who was not from the occupational therapy discipline and perceived this as a potential barrier to both the development of their occupational therapy professional identity and their future career progression. Having a nonoccupational therapy manager, which was identified as a common occurrence in the mental health context, at times led to a situation where a therapist's direct manager had a reduced understanding of the occupational therapy role. Subsequently, some participants noted this may lead to reduced opportunities compared to those provided by an occupational therapy manager. Participant 5 observed how this unfolded for a colleague:

She did not have the same opportunities that I had because she did have a line manager of a different discipline and did not quite understand occupational therapy as a discipline. I think that really limited her from progressing as quickly.

However, not all therapists had the same experience and the reflection may potentially relate to the quality of supervision and resources available to supervisors. For example, one participant with a nonoccupational therapy manager stated, “I think my direct line manager and I have similar ways of thinking and doing things and [we are from a] different discipline, but we do see things in a similar way, so I think that helps” (Participant 4).

##### 3.1.2.3. Individual Traits

Participants noted that their individual traits, such as being ambitious, self-directed, and engaging in reflective practice, could facilitate career progression. Ambition allowed participants to pursue new opportunities to develop and progress their careers. Participant 7 noted, “I feel like I probably always had a bit of a hunger to keep growing, keep developing, wanting to do more things.”

Other participants were able to reflect on and identify their learning needs to ensure they had clear plans for adequate support and skill development to progress. For example, Participant 1 noted

I highlighted that education is something that I was interested in, but my skill set around delivery of education I felt like I did not have confidence. I was able to flag that quite early on and get involved in actually facilitating some of the training.

Some participants identified that changing roles more frequently contributed to faster career progression. This style of working in several different roles in short spaces of time provided a varied experience to increase learning and competency. As Participant 2 noted, “I think a big part of the reason why I was able to move up quickly is because I changed jobs so many times”.

Participants, who were leaders and responsible for the development of staff, highlighted their inherent interest and desire to facilitate the career progression of others. Participant 5 gained satisfaction from this contribution, noting, “I think I also noticed that I really enjoyed developing other people within my team as well from an early time … I also see me helping the team, helps those consumers as well.”

#### 3.1.3. Perception of the New Career Framework

Within this broader category, participants discussed two main subcategories regarding how they perceived the new career framework for allied health staff being implemented in the local health district: (a) a good reference tool that provides structure and (b) limitations and ideas for implementation.

##### 3.1.3.1. A Good Reference Tool That Provides Structure

When provided with the career framework documents during their interview, all participants perused them and noted that they understood why it was created and its purpose. Some were familiar with the career framework, whilst others were aware of a new approach but were not as familiar, indicating that it was not yet widely utilised with the participant group. They could all see its relevance to career planning, development, and progression for mental health OTs.

Therapists could reflect on their career journey to compare their career progression and development to what was outlined in the career framework and see the similarities. For example, Participant 3 commented, “that's roughly what I did” after reviewing the career pathways in the framework. Participants also found the current methods of career progression in the health district less structured than the proposed framework, meaning that it was difficult to find information to facilitate progression prior to the career framework. Therefore, a new, more structured career framework was seen as potentially providing greater structure to those in mental health roles:

I think on my journey I feel like I've just stumbled across a lot of stuff. I do not feel like I had a very clear pathway … sometimes it felt like the hard way or the long way to get there. So yeah, I think it would be super useful. (Participant 7)

Participants emphasised the benefits the framework would provide in professional development reviews and supervision sessions, “especially with … clinical supervision it would be extremely helpful” (Participant 2). The career framework was described as “a good reference tool” by Participant 3 to navigate learning opportunities, determine skill requirements, and facilitate goal planning. It provided more structure to guide progression by identifying possible opportunities and educational courses available to staff to enhance career development. The visual diagrams and layout of the career framework document were also appealing, with one participant noting:

I think it would be really helpful actually because it's a visual way of showing where people are at and saying in discussion and supervision … “so this is where you're at currently and these are the things that you might need to work on to progress further.” That's a more structured way of doing it. (Participant 5)

##### 3.1.3.2. Limitations and Ideas for Implementation

Participants also identified limitations to the career framework's use for facilitating career progression for them specifically. As the career framework was designed to target most allied health professions, participants identified that its generalised nature somewhat limited the ability to explain the nuances of occupational therapy and lacked specificity related to mental health occupational therapy careers. This was noted as potentially limiting its use as a framework to facilitate individualised career progression. One participant noted, “I think it's hard when you get down to [the] specificit[ies] for each discipline and how each discipline will move forward across the life of an occupational therapist” (Participant 3). Therefore, participants suggested the career framework could be improved by ensuring that individualised career planning and progression could still occur when utilising the framework.

Other limitations to the career framework included the large number of pages contained in the document that may prevent some people from reading in its entirety; “the other thing I wonder about is the volume … I feel it's almost overwhelming, in terms of the breadth of what's here” (Participant 3). Upon perusing the framework document during the interview, one participant questioned terminology, highlighting an aspect of the framework related to “novice” clinicians. This participant pointed out that what was in the framework for this level of clinician did not align with their expectations of novice OTs:

To me this reads as though novice clinicians are typically underperforming, lacking confidence and not meeting basic competencies around safe practice. It seems quite harsh and I do not think this is necessarily supposed to read this way? My view is that new graduate clinicians should be demonstrating safe practice and meeting basic competencies and expectations, which is not how I am currently interpreting the way it is written. (Participant 6)

Finally, participants identified a missing link regarding how the health district was engaging their staff with the framework document. Participants agreed that it would be critical for the health district to facilitate staff engagement with the career framework to increase its use and maximise its potential to enhance career planning, development, and progression.

## 4. Discussion

This study provides insight into the career planning, development, and progression of mental health OTs within one large health district in New South Wales, Australia. This study explored the perspectives of career planning, development, and progression held by mental health OTs and how these align with a career framework created by the NBMLHD for its allied health staff. Whilst participants' perspectives aligned with each other, differing experiences and insights contributed to gaining a detailed description and understanding of mental health occupational therapy careers in this group.

Findings revealed that internal and external factors influenced career progression for this group. When emerging as novice therapists and embarking on their careers, participants discussed what influenced their choice of mental health career, such as a passion for working in the area and a positive placement experience, with these views ultimately impacting the rest of their careers. Furthermore, various factors, including secondments, supervision and management, and individual traits, were discussed as influential in the development of their careers. The participants discussed how current career guides and forums within the health district were less structured, resulting in a need to be proactive, with most “stumbling” upon courses and training to develop their careers. The participants highlighted the benefits of the newly introduced career framework as a positive step towards more deliberate planning and development, which may, in turn, benefit the career progression of those utilising this approach. Therefore, this study is the first of its kind to draw attention to deliberate career planning, development, and progression for mental health OTs and how a structured framework, utilised in nursing and allied health internationally, may benefit them.

Career progression for mental health OTs is multifaceted as each participant's career journey is unique and individualised. This aligns with findings from Nelson et al. [9] that the career progression of OTs, more broadly, is a nonlinear or sequential process. The current study participants discussed multiple avenues for career progression in mental health across different pathway modalities, client age groups, and settings that provide numerous opportunities for growth and development. The ability to work in both occupational therapy-specific and nondiscipline-specific roles increased job opportunities. However, participants discussed conflicting experiences with these roles, with the risk of reducing professional skills and identity. Furthermore, some participants perceived that challenges to career progression potentially arose when the therapist wanted to remain clinically focused as there were limited senior clinical role opportunities beyond Level 4. Additional support and guidance are required to maintain the occupational therapy workforce and navigate multiple progression avenues.

Starting a career in mental health was influenced by having a passion for working in the area and having a positive placement experience. A positive placement experience increased the therapists' understanding of the role of occupational therapy in mental health and changed their perceptions about pursuing a career in this area. For some participants, a passion for working in mental health was established in their formative years. This passion was observed to be a driving force for remaining in this area of practice. Although there is substantial literature surrounding the challenges of working in mental health as an OT [5–8, 27], the participants in this study did not comment on this when asked what influenced their careers. This may be attributed to the time the participants have been practising as OTs in the mental health area, whereby they had developed effective strategies to create a sustainable career. This suggests that having a passion for working in this area may assist in reducing the burden of challenges encountered.

Once embarking on their careers, the participants identified and discussed how secondments, supervision, and management influenced their career progression. These factors were addressed by most of the participants; however, not all perspectives aligned. For instance, the participants all discussed how pivotal secondments were to career development; however, if not planned or executed effectively, they created additional stress and an expectation to keep both roles afloat at the same time. This adds to Craik [10], who discussed the role secondments have in exploring opportunities for future growth and progression. Findings from this study further emphasise the benefits for both the individual and wider organisation by offering the opportunity to temporarily develop skills and experience in a new role whilst solving staff shortages for the service.

Similarly, with supervision, participants identified the pivotal role this held in facilitating progression throughout the entirety of their occupational therapy mental health career. Aligning with findings from Nelson et al. [9], this study also provides additional perspectives on the conflicting experiences regarding having a manager not from the occupational therapy discipline acting as either a facilitator or barrier to progression for some. Furthermore, individual traits such as ambition, self-direction, and reflection were also influential in career progression for mental health OTs. Supported by findings in previous studies [10, 12], these traits allowed for new opportunities to be obtained to meet personal goals and further their career. This study provides the perspectives of senior mental health OTs and highlights how these traits are valuable to career progression and securing higher-level positions. Additionally, this study reveals the impact frequently changing roles can have on career development. This working style developed broad skill sets, experience, and expertise in a timely manner, helping to achieve competencies that enabled career progression.

The mental health OTs in this study acknowledged the health district's efforts to create an allied health career framework. They appreciated its intended purpose of supporting staff with career progression. They viewed this as a positive and much-needed step to support staff in a structured way, offering a central point of information that was previously less structured. The career framework is one of a few career frameworks that have recently been published to expand Benner's model beyond nursing and address career planning for allied health professionals. To the knowledge of the research team, this research is the first of its kind to examine how this new strategy of supporting career planning, development, and progression, in the form of a framework, aligns with the perspectives of allied health staff who are its focus. As such, it provides a unique opportunity to address how current careers progress for mental health OTs in the health district, what influences these careers, and the extent to which this new framework aligns with these experiences.

Some participants discussed how the levels of progression and competencies in the framework mirrored their career journey and the benefits of this career framework in facilitating supervision, performance reviews, and career development plans. However, despite the framework being seen as a good reference tool that provides structure to career planning, the participants identified limitations. Participants expressed concern that the framework may not be sufficiently specific to explain the nuances of mental health occupational therapy careers due to it being targeted to all allied health professions within the district. Additionally, participants were concerned that others might view the career framework as a step-by-step instruction rather than a guide, thus imposing a linear structure on the typical nonlinear progression of occupational therapy careers [9, 27], which may in turn result in missed opportunities. Participants also expressed the need for the health district to better engage the allied health group with the career framework, specifically the mental health occupational therapy team. In doing so, it was hoped that the framework would reveal identified work role challenges, which could be addressed to retain and support mental health careers within the district. Therefore, this study provides positive insight into how Benner's model applies to the career progression of mental health OTs as the participants' experiences resonated with the competency levels presented in the framework (novice, beginner, adept, advanced, and senior specialist). As this is the first research of its kind, further research should be carried out with other OTs and allied health professionals in the health district to determine the framework's ability to support the career development of these groups.

## 5. Limitations

The study sample is small and restricted to the lived experience of senior OTs working in the mental health service within one specific local health district. Whilst the current participants recalled and reflected on their own career planning and progression from novice to expert, we could not recruit new and emerging clinicians (*N* = 10), and this is a major limitation of our study, especially in understanding how the current novice–beginner planned to transition to more senior roles, and or why they left the profession or discipline area or workplace. Future studies focusing on the career intention, planning, and development of merging mental health OTs would shed unique insights into workforce retention. Furthermore, all participants have been with the local health district for an average of 10 years; this may also limit the ability to generalise the findings to clinicians who have left the practice area (mental health) quite soon after entering the occupational therapy workforce. Therefore, the findings are specific to this group and its context. However, other health districts and international readers may find the findings relevant, given that they are discussed in relation to the broader area of mental health occupational therapy practice. Therapists working in the public health system may experience career pathways similar to those of the participants in this study.

Two different interview modes were used: face-to-face and online. This did not appear to affect the information provided by participants, as rich descriptions of their unique experiences were able to be captured, and interviews flowed smoothly under both modes.

## 6. Conclusion

This study has provided new information pertaining to career planning, development, and progression of OTs working in a mental health setting. Findings align with previous studies highlighting the multifaceted nature of career progression in this important area of practice. Influencing factors provide valuable information on how to best support and retain occupational therapy staff. The NBMLHD allied health career framework offers new insight regarding how Benner's (1982) model can be utilised to support the career progression of mental health OTs and facilitate their career development.

## Figures and Tables

**Table 1:** Overarching category, categories, and subcategories.

**Overarching category**	**Category**	**Subcategory**
Mental health occupational therapy career progression is multifaceted	Influences on the choice of a mental health career	Passion for working in mental health
		A positive placement experience
	Influences on career progression	Secondments
		Supervision and management
		Individual traits
	Perception of the new career framework	A good reference tool that provides structure
		Limitations and ideas for implementation

## Data Availability

The data supporting this study's findings are available from the corresponding author, D.L., upon reasonable request.

## Supporting Information

Supporting Information Additional supporting information can be found online in the Supporting Information section. Allied health level pursuant to the New South Wales Health Service Health Professionals (State) Award 2024.
