# Supplementary material for: Occupational Therapists' Career Planning, Development, and Progress: An Australian Mental Health Perspective
Source: Occup Ther Int. 2025 Feb 26;2025:3901634. doi: 10.1155/oti/3901634 (PMC11985222; doi:10.1155/oti/3901634)
Supplement: Supporting Information — Additional supporting information can be found online in the Supporting Information section. Allied health level pursuant to the New South Wales Health Service Health Professionals (State) Award 2024. [file 3901634.f1.docx]

Supporting Information: Allied health level pursuant to the New South Wales Health Service Health Professionals (State) Award 2024

Level 1: newly qualified allied health professionals who are beginning practitioners developing their skills and competencies.

Level 2: competent, independent practitioners with at least 3 years of clinical experience.

Levels 3 and 4: experienced clinicians who possess extensive specialist knowledge of a high level of broad generalist knowledge, have advanced reasoning skills, and provide clinical services of a complex nature requiring advanced practice skills.

Levels 5 and 6: may have a clinical, education or management focus or elements of all three features. Roles include department head, unit head/ team leader, health professional educator, clinical specialist (with postgraduate qualification or fellowship)

Level 7: managers, leaders, or managers of large units, teams or departments

Level 8: Discipline-specific director that leads, direct, coordinates and provide strategic advice on major functions or areas within a local health district

For more information about the education and qualifications of respective occupational therapist level, please see: https://www.nsw.gov.au/employment/my-career-planner/explore-occupations/occupational-therapist
